# Supplementary material for: Abundance of the Dominant Endosymbiont Rickettsia and Fitness of the Stored-Product Pest Liposcelis bostrychophila (Psocoptera: Liposcelididae)
Source: Insects. 2025 Mar 27;16(4):349. doi: 10.3390/insects16040349 (PMC12028104; doi:10.3390/insects16040349)
Supplement: Supplementary file 1 [file insects-16-00349-s001.zip › insects-3454418-supplementary.pdf]

Supplementary Material

Quantification of Rickettsia

DNA Extraction

Genomic DNA from *L. bostrychophila* at different developmental stages and under different temperature conditions was extracted using the Animal Genomic DNA Rapid Extraction Kit (B518221). The purity and concentration of the DNA were measured using a microvolume spectrophotometer (Merinton Instrument, SMA4000). The extracted DNA, which met the required quality standards, was stored at -20 ℃ for the next step.

Standard Preparation

1）Using the extracted DNA as a template, PCR amplification of the target gene fragment was performed with *Rickettsia* specific primers (*Rb*-F 5′-GCG-GATTGTTTTCTAGCTGTC-3', *Rb*-R 5'-TGGGTTTCGGTCATCGTGT-3'). The specific primers used in this study were designed by us. During the design process, key parameters such as primer length, GC content, and annealing temperature were taken into account. After the design was completed, we verified the specificity of these primers through melt curve analysis in quantitative PCR. Experimental results showed that these primers could specifically recognize the target sequences during amplification, ensuring the reliability and accuracy of the experiment. The PCR reaction mixture (see Table S1) was used with the following program: 95 ℃ for 3 minutes for initial denaturation; then 94 ℃ for 30 seconds for denaturation, 57 ℃ for 30 seconds for annealing, and 72 ℃ for 30 seconds for extension; followed by a final extension at 72 ℃ for 8 minutes. The reaction was carried out for 35 cycles. The PCR amplification results were analyzed using 1.5% agarose gel electrophoresis. The target bands were recovered from the gel using the SanPrep Column DNA Gel Extraction Kit (B518131).

Table S1. Reaction system for PCR.

Reagents	Volume(μL)
10X PCR Buffer	2.0
upstream primer(10 μm)	0.5
downstream primer(10 μm)	0.5
dNTP（10 mM）	0.5
$MgCl_2$ (25 mM)	2.0
Taq Plus DNA Polymerase（5 U·μL^{-1}）	0.5
DNAtemplate	2.0
ddH_2O	17.0

2）The target gene fragment was ligated with the vector using the pMD® 18-T Vector Cloning Kit (TAKARA, 1000328), and transformation was performed with the Fast Competent Cell Preparation Kit (B529307). After transformation, plasmid DNA was extracted using the SanPrep Column Plasmid DNA Mini-Prep Kit (B518191). The plasmid was then sequenced for identification using an ABI sequencer (Foster, CA, USA).

3）After the constructed plasmid was verified by sequencing, the OD_{260} value of the plasmid was measured using a microvolume spectrophotometer. The plasmid copy number (PCN) was calculated using the following formula (S1):

$$\text{PCN (copies}\cdot\mu L^{-1}) = [\text{plasmid concentraion}(\text{mol}\cdot\mu L^{-1})] \times 6.02 \times 10^{23}$$

$$= \frac{[\text{plasmid concentraion}(g\cdot\mu L^{-1})]}{M} \times 6.02 \times 10^{23}$$

$$= \frac{[\text{plasmid concentraion}(\text{ng} \cdot \mu\text{L}^{-1}) \times 10^{-9}]}{\text{M}} \times 6.02 \times 10^{23}$$

$$= \frac{[\text{plasmid concentraion}(\text{ng} \cdot \mu\text{L}^{-1})]}{\text{M}} \times 6.02 \times 10^{14} \qquad \text{(S1)}$$

where M was molecular weight, the value of M was calculated by using the following equation (S2):

M=the base number of recombinant plasmids（bp）×650 $(\text{g} \cdot \text{mol}^{-1})$ = [the base number of vector (bp) + the base number of PCR product (bp)] ×650 $(\text{g} \cdot \text{mol}^{-1})$ (S2)

The average molecular weight of one DNA base pair (sodium salt) = 650 daltons.

The constructed plasmid was subjected to a 10-fold serial dilution (90 μl dilution buffer + 10 μl plasmid). Plasmids with concentrations ranging from 10^2 to 10^7 copies·μL^{-1} were used as standard templates for TaqMan qPCR amplification. A standard curve was established by plotting the Ct value on the vertical axis and the logarithm of the standard concentration on the horizontal axis.

Fluorescent Quantitative PCR Detection

The reaction mixture for TaqMan qPCR was prepared using the 2X TaqMan Fast qPCR Master Mix (B639274, BBI, Roche) as described in Table S2. TaqMan qPCR amplification of the extracted DNA was performed using the LightCycler480 II fluorescence quantitative PCR instrument (Roche, Rotkreuz, Switzerland). The qPCR program was set as follows: 94 ℃ for 3 minutes for initial denaturation; 94 ℃ for 5 seconds for denaturation, 57 ℃ for 15 seconds for annealing, and 72 ℃ for 30 seconds for extension, with 45 cycles. The primers and probe used were: *Rb*-F 5′-GCGGATTGTTTTCTAGCTGTC-3′, *Rb*-R 5′-TGGGTTTCGGTCATCGTGT-3′, and *Rb*-P 5′-FAM-TGCCGCAGTACTTA-MGB-3′.

Table S2. Reaction system for qPCR.

Reagent	Volume(μL)
TaqMan Fast qPCR Master Mix (2×)	5.0
upstream primer(10 μm)	0.2
downstream primer(10 μm)	0.2
probe(10 μm)	0.2
DNA template	1.0
ddH_2O	3.4
